# Classification of Cattle Behaviours Using Neck-Mounted Accelerometer-Equipped Collars and Convolutional Neural Networks

**DOI:** 10.3390/s21124050

**Published:** 2021-06-12

**Authors:** Dejan Pavlovic, Christopher Davison, Andrew Hamilton, Oskar Marko, Robert Atkinson, Craig Michie, Vladimir Crnojević, Ivan Andonovic, Xavier Bellekens, Christos Tachtatzis

**Affiliations:** 1 BioSense Institute, 21101 Novi Sad, Serbia; oskar.marko@biosense.rs (O.M.); crnojevic@biosense.rs (V.C.); 2 Department of Electronic and Electrical Engineering, University of Strathclyde, Glasgow G1 1RD, UK; christopher.davison@strath.ac.uk (C.D.); andrew.w.hamilton@strath.ac.uk (A.H.); robert.atkinson@strath.ac.uk (R.A.); c.michie@strath.ac.uk (C.M.); i.andonovic@strath.ac.uk (I.A.); xavier.bellekens@strath.ac.uk (X.B.); christos.tachtatzis@strath.ac.uk (C.T.)

**Keywords:** precision agriculture, convolutional neural networks, cattle behaviour monitoring

## Abstract

Monitoring cattle behaviour is core to the early detection of health and welfare issues and to optimise the fertility of large herds. Accelerometer-based sensor systems that provide activity profiles are now used extensively on commercial farms and have evolved to identify behaviours such as the time spent ruminating and eating at an individual animal level. Acquiring this information at scale is central to informing on-farm management decisions. The paper presents the development of a Convolutional Neural Network (CNN) that classifies cattle behavioural states (‘rumination’, ‘eating’ and ‘other’) using data generated from neck-mounted accelerometer collars. During three farm trials in the United Kingdom (Easter Howgate Farm, Edinburgh, UK), 18 steers were monitored to provide raw acceleration measurements, with ground truth data provided by muzzle-mounted pressure sensor halters. A range of neural network architectures are explored and rigorous hyper-parameter searches are performed to optimise the network. The computational complexity and memory footprint of CNN models are not readily compatible with deployment on low-power processors which are both memory and energy constrained. Thus, progressive reductions of the CNN were executed with minimal loss of performance in order to address the practical implementation challenges, defining the trade-off between model performance versus computation complexity and memory footprint to permit deployment on micro-controller architectures. The proposed methodology achieves a compression of 14.30 compared to the unpruned architecture but is nevertheless able to accurately classify cattle behaviours with an overall F1 score of 0.82 for both FP32 and FP16 precision while achieving a reasonable battery lifetime in excess of 5.7 years.

## 1. Introduction

Sensor-based cattle behaviour monitoring has been driven by the need to optimise herd fertility and improve animal welfare, both leading to increased production yields. Economic pressures on the sector have resulted in the consolidation of small scale cattle farming concerns. For example, in the UK, the number of milk producers has fallen from over 35,000 in 1995 to under 12,200 in 2019 and the number of dairy cows from over 3.2 million in 1980 to under 1.9 million in 2019. During the same period, milk production has slightly increased—13,320 M L in 2008 to 14,960 M L in 2020 [1]. The increase in productivity has been secured through improvements in genetic gain, but also through the adoption of precision agriculture technologies [2,3]. An ever-increasing range of measurement/monitoring devices and systems are commercially available, providing dairy farmers with information, for example through alerts, on the early onset of health issues and highly accurate identification of the onset of oestrus (or ‘heat’), both integral to optimising milk yield per animal [4,5,6]. Systems such as neck-mounted collars, leg and ear tags are now in common use to monitor dairy and beef cattle, providing outputs that inform farmers on the most appropriate and targeted management interventions [6,7,8].

One of the most insightful behavioural characteristics for assessing cattle welfare and fertility is the time spent ruminating, the process during which the animal regurgitates and masticates previously ingested food to aid the digestion process and improve nutrient absorption [6,9,10]. The time spent ruminating is a key indicator of health as cattle that are ill or injured eat less and thus ruminate less; therefore estimating the time spent ruminating is critical information for effective herd welfare management. Moreover, the detection of oestrus is essential to optimising herd fertility as the accurate identification of the optimum window for cattle to be inseminated improves pregnancy rates and in turn increases milk production. A missed oestrus cycle has a significant impact on yield, dependent on the region and established management practices. For example, the lost revenue from a missed heat event is in the region of GBP 120 based on current UK farm gate average price of GBP 0.286 per litre and a daily production of 20 L [11]. The onset of oestrus is also accompanied by other changes in behaviour such as a drop in the time an individual animal typically spends ruminating. Accelerometer-based sensor systems provide a means for observing and classifying a range of behaviours continuously, carrying out time consuming tasks traditionally executed through visual inspection.

The objective of the study was to examine the feasibility of utilising Deep Learning (DL) Neural Network approaches to detect multiple cattle behaviours using neck-mounted accelerometer-equipped collars with low-cost, low-power computationally constrained micro-controllers. The proposed framework initially builds a CNN that eliminates the burden of the feature engineering process that typically accompanies traditional Machine Learning (ML) approaches, and it is often difficult to introduce new behaviour states in the predictive model. The full CNN is subsequently pruned to minimise the memory, computation and consequently energy demands without sacrificing classification performance. These elements make CNN approaches practical for deployment on resource-constraint micro-controller devices and permit their adoption in on-farm/on-animal applications.

## 2. Related Work

Table 1 summarises solutions reported for the identification of cattle behaviours utilising a range of devices and machine learning algorithms. Furthermore, Table 1 presents the data set sizes and the ground truth methodology used to validate each solution. In some cases, the description of the data sets is ambiguous and thus unknown parameters are represented by a ‘-’. Additionally, a performance comparison in terms of Accuracy, Precision, Recall and F1 score (where these have been reported) between the literature and this study is shown within Table 1. It is important to note that it is hard to make direct comparisons between studies as some report the performance of their best model while others a range of models, sometimes for individual behaviours or aggregate across behaviours. Table 1 lists the metrics of the best model or the performance range where these are available. Even when the reported performance metrics are identical, it is unclear if the performance difference can be attributed to differences in animal breeds and/or farming practise, data set size and ground truth methodology, algorithmic differences, evaluation protocol, behaviours, number of behaviours, device or device location. Furthermore, the definition of behaviour differs between studies (Table A1), for example, Grazing [12]/Feeding [13]/Eating [14] or Moving [15]/Walking [16]/Travelling [17]. To mitigate these issues, the current study makes the data set publicly available to permit the community to perform direct algorithmic comparisons. Furthermore, the data set size is comparable to that of other research reported in the literature in terms of the number of animals, while the total number of observation hours is significantly higher (the data set is publicly available at https://www.doi.org/10.5281/zenodo.4064801 (accessed on 9 June 2021)).

A number of classical machine learning algorithms approaches such as Support Vector Machines [10,13,22] and Decision Trees [17,18,24] have been used to classify cattle behaviours. However, these approaches required considerable effort to extract features from sensor signals that permit accurate discrimination between behaviours. Feature extraction is often a time-consuming and highly complex process, requiring a contribution from domain knowledge experts with many years of on-farm operational experience. Moreover, despite the fact that such features are effective in discriminating the targeted behaviours, they are not extensible to additional behavioural classes; new classes demand the engineering of new features. An approach that reduces the challenge inherent to manual feature engineering utilises auto-encoders [16] that automate the feature extraction process prior to the Support Vector Machine classifier. Here, data generated by 3-axis accelerometer neck-mounted collar sensors were used to classify nine cattle behaviours. The data set from the collars was acquired from 22 animals over a period of 8 days, and each animal was also directly observed by humans over two hours at least once during three daily observational periods. ‘Ground truth’ data, central to algorithm development, are often obtained through direct animal observations [12,13,16,17,21] or through video annotations [13,15,18,22,23,24] executed by animal scientists. Both methods require significant effort over long periods of time, and as a consequence the ground truth sets are relatively small, comprising no more than several days of data. Moreover, the recent adaptation of Deep Learning approaches for cattle behaviour classification has dramatically increased the demand for larger data sets.

An alternative automated approach to ‘ground truth’ data generation is muzzle-mounted halter pressure sensors enabling continuous acquisition without human intervention. The halter measures the jaw movements of an animal directly through concomitant changes in pressure of a strap around its muzzle, in so doing classifying behaviours such as rumination and eating [26]. The halter is an accepted means of gathering ground truth data since it has been shown to yield excellent correlations between the measured time spent ruminating and eating with these times obtained through human observation. Studies [14,27] have reported a high Spearman correlation of 0.96 and 0.75 for rumination and 0.96 and 0.81 for eating, respectively. Furthermore, a similar study that utilised video annotations [23] obtained a F1 Score of 0.932 for rumination. However, the halter is not compatible with production settings since it is expensive, has a short battery lifetime and is intrusive (full face harness and muzzle).

A study utilising a Deep Learning approach for cattle behaviour classification [21] details the use of a Convolutional Neural Networks (CNNs) for the identification of grazing and non-grazing periods. The data used within the study are as in [16] but given that the target in [21] is binary, the classification is less challenging compared to multi-state behaviour identifications. A Recurrent Neural Network with Long Short-Term Memory (RNN-LSTM) has been demonstrated to classify eight behaviours such as ‘feeding’ ‘ruminating’, ‘licking salt’, ‘social licking’ and ‘head butting’ using data generated by a combination of a 3-axis accelerometer/gyroscope/magnetometer as inputs to the classifications [15]. Two cameras were used to record cattle behaviours over 7 days, providing a highly appropriate validation set for the development of the algorithms. The RNN-LSTM framework yielded accurate classifications, but its operational deployment in low-cost embedded hardware characteristic of practical on-farm deployments, remains a challenge owing to significant algorithm complexity.

In the work presented in the current study, the development of a Machine Learning framework for the classification of multiple animal (cattle) behaviours based on CNNs using 3-axis accelerometer data is reported and the performance of the network is evaluated. A Deep Learning approach is adopted to allow features to be learned automatically from raw accelerometer data, eliminating the burden of feature engineering/discovery. Similar to Recurrent Neural Networks (RNNs), CNNs are complex models and consequently not readily compatible with low processing power deployment. Thus, an examination of the trade-off between performance and model size (memory footprint and computational complexity) to enable the engineering of a solution that can be implemented using low-cost, low-complexity processors that do not consume significant levels of power is carried out.

## 3. Materials and Methods

**Figure 1:**
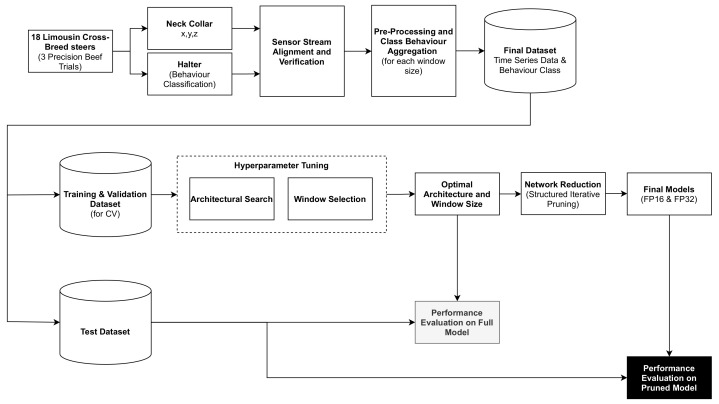
A block diagram showing all stages of the proposed methodology.

A block diagram detailing the development process, which comprised data acquisition, data pre-processing, hyper-parameter search, network reduction and performance evaluation is illustrated in Figure 1.

**Figure 2:**
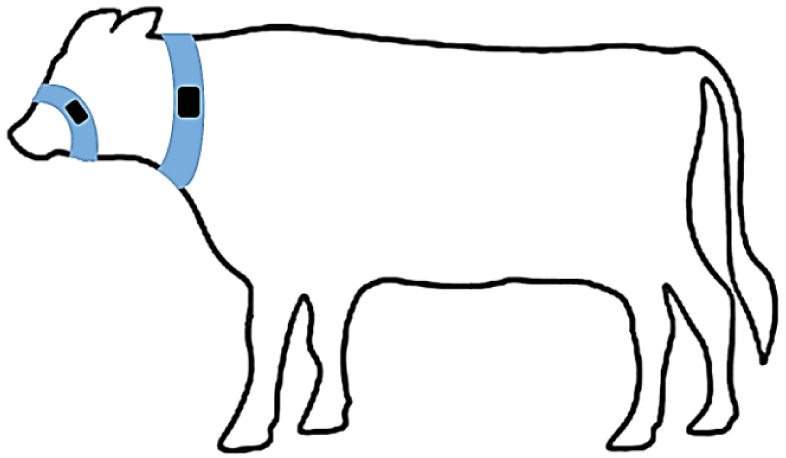
Placement of Rumiwatch halter on steer muzzle, Afimilk Silent Herdsman collar around animal’s neck.

**Figure 3:**
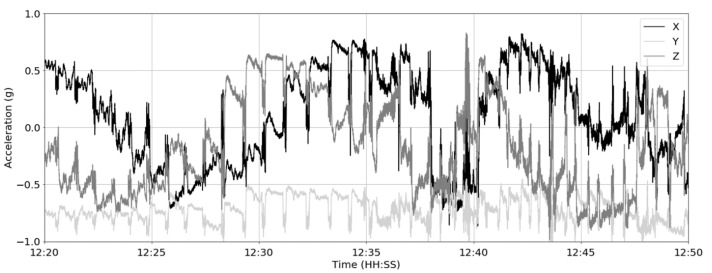
Example of raw 3-axis accelerometer data over a 30 min window at 10 Hz sampling frequency.

A series of data gathering exercises was conducted on three farm trials in the United Kingdom (Easter Howgate Farm, Edinburgh, UK) to enable the development and evaluation of the performance of the proposed CNN trained on accelerometer-derived data from neck-mounted collars. Neck-mounted collars are able to capture ‘rumination’, ‘eating’ and ‘other’ behaviours from measurements of the overall animal movement and from contractions of neck muscles [28]. A total of 18 Limousin Cross-Breed steers from three trials conducted in the period of June 2015 to October 2016 were equipped with Afimilk Silent Herdsman [5] and Rumiwatch halters [26], the collar mounted on the neck and the halter on the muzzle (Figure 2). The collar comprised a 3-axis accelerometer, an SD card for data storage, and a Real Time Clock (RTC), whilst the halter consisted of a pressure sensor, an SD card and RTC (both systems operated at a sampling frequency of 10 Hz). The collars provided acceleration values orientated in *x*-, *y*- and *z*-directions capturing both head and neck muscle motions (Figure 3), whilst the halter, through pressure changes induced by movements of the jaw, provided the ground truth of the following animal states:

- Rumination—the animal regurgitates partially digested feed, which is re-chewed and re-swallowed, aiding the further breaking down of the feed and thus improving nutrient absorption.
- Eating—the animal is ingesting food from a feed source.
- Other—the animal is engaged in activity which is neither ruminating nor eating.

**Figure 4:**
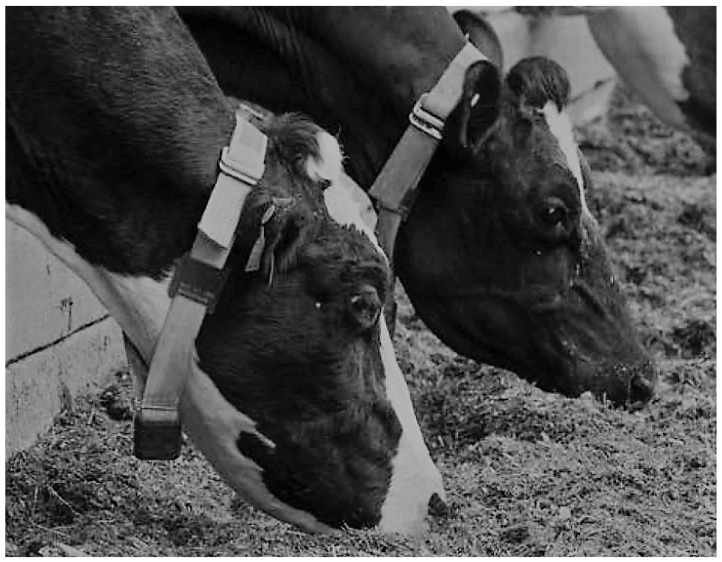
Activity collars on cattle.

Although every attempt was made to mount collars in identical positions around the necks of individual steers, differences in the anatomy between animals and continuous motion result in collars shifting and rotating. Although a weight is positioned on the bottom of the collar (Figure 4) in an effort to maintain a constant collar position, residual time varying biases are created because of orientation of the accelerometer with respect to the gravitational field. To eliminate these offsets and capture only the accelerations due to animal motion, the discrete difference along each axis is computed as;

(1) Δs[t]=s[t]−s[t−1], ∀s∈{x,y,z}

where s[t] is the raw acceleration signals for all axes (*x*, *y* and *z*), Δs[t] is the resultant signal for a given axis at time step *t*.

The steers were housed indoors on a straw bedding and fed a Total Mixed Ration (TMR) ad libitum. A total of 3460 h of data were acquired from both sensor systems and verified for time alignment. After the study was completed, both the collars and halters were removed and the data from their SD cards were recovered; both streams were merged by timestamp into unified data sets per animal as ‘.csv’ files.

## 4. Results and Discussion

### 4.1. CNN Design and Performance

**Figure 5:**
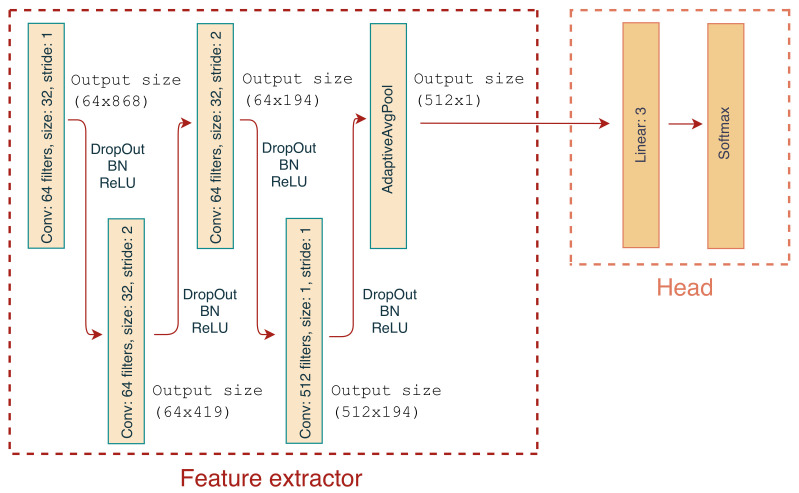
1D CNN Architecture consisting of a feature extractor and head segments.

The classifier architecture was based on a multi-class CNN that takes as an input three time series segments (one for each axis x, y and z) and outputs the predicted class. The basic architecture consists of two logical blocks as shown in Figure 5; feature extractor and head. The feature extractor consists of 4 blocks of convolutional *Dropout* [29], *BatchNorm* [30] and *ReLU* [31] layers. The kernel size and strides for all layers is shown in Figure 5. Note that in the last layer, the kernel size is 1 which permits the expansion of the feature map from 64 to 512. Finally, the extractor contains an Adaptive Average Pooling layer to aggregate features on the spatial dimension and create a consistent output size for cases where the input length varies, necessary to allow exploration of the impact of window size on performance described in Section 4.1.3. The head consists of a single fully connected layer with 512 inputs and 3 outputs, followed by *Softmax* to produce the classified output.

#### 4.1.1. Training and Validation

Initially, the accelerometer data were segmented into 90 s blocks (note, that the proposed system is evaluated on various window lengths in Section 4.1.3) with each data block labelled as one behaviour state only for truthing. Considering that the halter provides a label at a frequency of 10 Hz there are instances the truthing data indicates more than one behaviour state during the 90 s block, e.g., ‘eating’ with a transient shift into ‘rumination’. For these instances, a majority vote was applied within each block to indicate the primary behaviour. Naturally, each steer spends varying amounts of time in each of the behaviour states and for that reason, each steer data set was stratified to yield a balanced (equal) representation of each behaviour.

The resulting data were then divided into two sets, one for training/validation, the other for testing. Three complete steer traces, one from each farm trial, were selected randomly to create the test set. The remaining 15 steer traces are then used for training and validation. The training/validation data set was sliced using 5-fold cross-validation for each animal. The validation process was achieved by inserting all the unique steer identifiers in a vector, shuffling the vector and slicing into 5 folds. Thus, each fold contained 3 animals, in effect resulting in 12 animals in the training set and 3 in the validation set for each of the 5 splits. Every animal is included once in the validation set and the other four times as a compound of the training set. Finally, in order to ensure that there are no biases from individual animals, the training set was stratified to include equal representation from all 12 animals.

The network was trained with AdamW optimiser [32] at a learning rate of 1 × 10^−4^ utilising a one-cycle training policy [33] and cosine learning rate annealing. The batch size was set to 256. The network was trained to a maximum of 50 epochs with early ending when validation loss reached a plateau [34] at a patience of 15 epochs and delta change 0.01. Furthermore for all results, a weight decay at a λ of 0.01 was used. The optimum model is selected from the training cycle after the completion of the process.

The F1 score was used to evaluate CNN performance, defined as the harmonic mean of precision and recall as;

(2) $$F1=2\times \frac{precision\times recall}{precision+recall}$$

where precision and recall are defined as

(3) $$precision=\frac{True\;Positives}{True\;Positives+False\;Positives}$$

(4) $$recall=\frac{True\;Positives}{True\;Positives+False\;Negatives}$$

A True Positive (TP) is defined as an instance where one of the classes is correctly identified; a False Positive (FP) is a prediction of a certain class during a period where the steer was not in that behavioural class; a False Negative (FN) identifies a case where a period of a certain activity was incorrectly judged as some other activity.

#### 4.1.2. Hyper-Parameter Tuning

**Figure 6:**
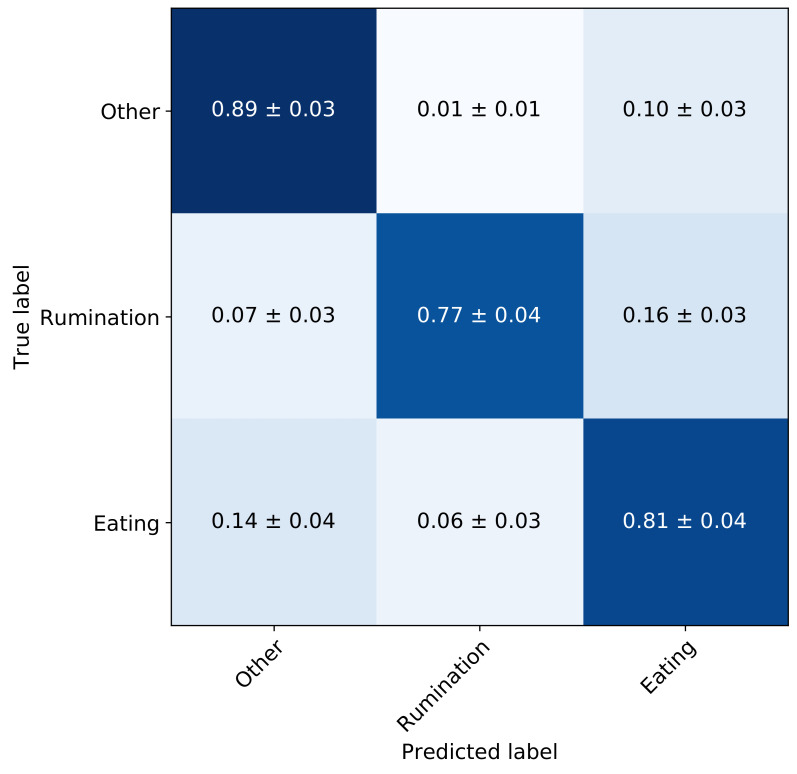
Confusion matrix of the full network after hyper-parameter tuning—validation set.

A hyper-parameter search was performed to optimise the number of convolutional blocks in the feature extractor, the kernel size of the convolutional filters and the probabilities of the *Dropout* layers. The number of filters for the feature extractor is shown in Figure 5 which remained constant throughout the hyper-parameter search. Considering the stochastic nature of model training, the process was repeated 5 times and the model with the highest average F1 score across all 5 folds was selected. A CNN with four convolutional blocks, kernel size of 16 and *Dropout* probability of 0.25, yielded the best performance. The mean validation performance for these parameters was a F1 score, precision and recall of 0.82, 0.83 and 0.82, respectively. The average validation confusion matrix is shown in Figure 6 along with the standard deviation for all cells. For instance, the normalised TP performance for ‘eating’ is 0.81 ± 0.04, while ‘rumination’ is mis-classified as ‘eating’ 0.16 ± 0.03. In all cases the standard deviation is below 0.04. Assuming the average time spent ruminating is around 400 min per day, an increase in sensitivity of 1% would result in an increase of around 4 min of time spent ruminating daily.

**Figure 7:**
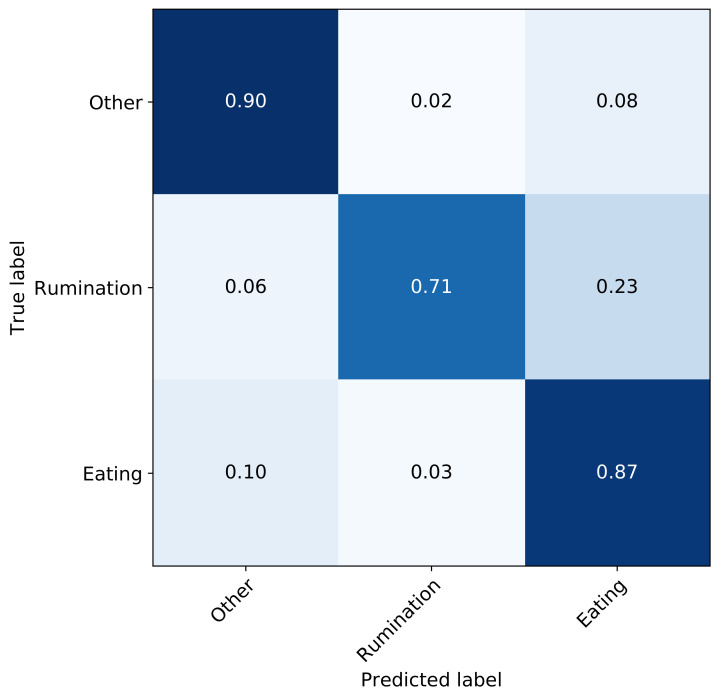
Confusion matrix of the full network after hyper-parameter tuning—test set.

The model with the best performance on the validation set was then selected for evaluation on the test set. The performance of the validation set was F1 score, precision and recall of 0.86, 0.87, 0.86, respectively, while the test set F1 score, precision and recall of 0.82, 0.84, 0.82, respectively. Finally, the confusion matrix on the test set is shown in Figure 7. Since ‘eating’ and ‘rumination’ are characterised by similar jaw motions, the model suffers the most confusion between these two states; the confusion between other states is significantly lower.

For the full test data set, without stratification or balancing (i.e., all the data from all three test steer traces), the weighted performance metrics F1 score, precision and recall were 0.82, 0.87, 0.81, respectively.

#### 4.1.3. Window Lengths

The definition of the classification window is essential for the practical implementation of animal behaviour classification, i.e., how frequent should the classifications be performed. Behaviour varies from animal to animal and certain behaviours occur for a few seconds whilst others typically last a few minutes. For instance, rumination contractions typically occur at 40–60 s intervals, while sudden head movements are in relation ‘instantaneous’. Thus, the selection of an inappropriately time-restricted classification window will lead to higher granularity of the classification, resulting in missed behaviours and increased classifier confusion (behaviours which are captured incompletely). Conversely, a window length which is excessively large leads to coarser classification and each time window will contain multiple behaviours in turn increasing classifier confusion. Therefore an evaluation of the sensitivity of the classifier performance of the proposed network architecture as a function of window lengths was carried out. Furthermore, a classifier trained on data of a predefined window length, can be used to classify behaviours of other lengths given that the last layer of the feature extractor in Figure 5 implements Adaptive Average Pooling.

**Figure 8:**
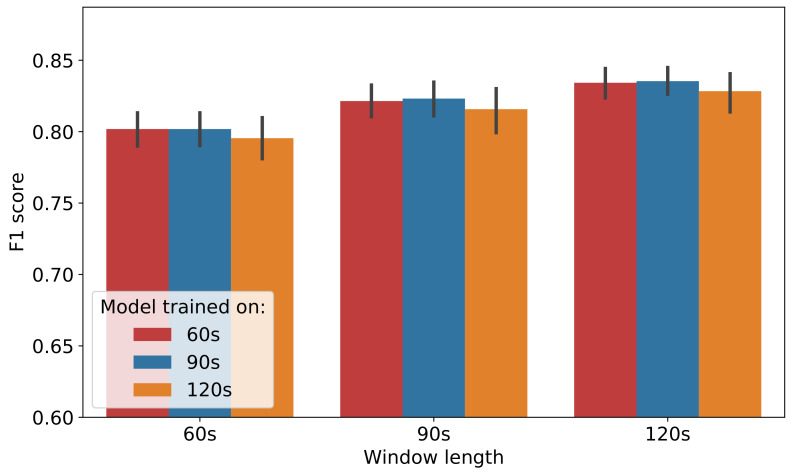
Performance of models trained and evaluated on different window lengths.

Figure 8, shows the F1 score performance of models trained on 60 s, 90 s and 120 s window lengths and evaluated on 60 s, 90 s and 120 s data. The bar heights represent the average F1 score for the 5-fold cross-validation and for 5 random repetitions for the corresponding models obtained after hyper-parameter tuning (described in Section 4.1.2), while the error bar represents the 95% confidence interval (the Confidence Interval is computed with boot-strapping [35]) of the performance. Evident is that models trained on 120 s data yield lower performance compared to 60 s and 90 s owing to the severe aggregation of behaviours in 120 s data. The 60 s and 90 s models exhibit almost identical performance, however the 60 s model has lower memory footprint, computational complexity and hence is preferred. It should be noted that the above window size is at odds with the current window length utilised by the commercial collar system such as the Afimilk Silent Herdsman [5] and for that reason, the remainder of the evaluation utilises models with window length of 90 s.

### 4.2. Network Reduction

Although the proposed CNN offers a high performance for the classification of steer behaviours, the model size in terms of computation complexity and memory footprint is a significant barrier to deployment on low-cost, low-power micro-controllers, typical in commercial neck-mounted collar implementations. Furthermore, on-farm sensor systems rely on battery power and high energy consumption applications limit device lifetime rendering solutions impractical.

A number of techniques have been proposed to reduce memory and energy consumption requirements of neural network models, such as weight low-rank approximation [36,37], knowledge distillation [38], weight quantisation [39,40,41] and network pruning [42], all with the proviso that any approach must not compromise network performance significantly. Here, although network pruning is evaluated in the goal to reduce the network size and computational overhead, the other methods could be utilised in tandem to provide further reductions. Broadly speaking, network pruning can be categorised as structured and unstructured; for the latter [43,44,45,46], individual parameters with low significance are removed from the network and although the technique increases sparsity, this does not necessarily result in memory or computation benefits. Unstructured pruning is often implemented by zeroing the weights, however these weights still exist in the parameters matrices occupying memory and consuming computations. On the other hand, structured pruning [47,48,49,50] allows the elimination of complete segments of the parameters matrices resulting in benefits in respect of both memory storage and computation, making models more amenable to deployment on micro-controllers.

The impact of structured pruning on the performance of the CNN has been evaluated under the principle that neurons with small weights are less significant and filters with the smallest weights are the most likely candidates for pruning. An example is the use of convolutional filter importance—determined using $\ell_{1}$-norm, i.e., the sum of its absolute weights—with one-shot pruning [47], although it is acknowledged that iterative pruning may yield an improvement in performance. Here, the pruning was performed over multiple stages where each stage contains both an iterative pruning and fine-tuning phase. The initial pruning phase is applied to the optimal network architecture discovered through the hyper-parameter search stage described in Section 4.1.2 and the number of filters in all convolutional layers was set to 64.

Pruning is performed gradually within each pruning iteration following the schedule defined by Equation (5) [51]. The sparsity $s_{t}$ at each iteration (epoch) *t* is;

(5) $$s_{t}=s_{f}-s_{f}\times {\left(1-\frac{t}{n\Delta t}\right)}^{3},\;t\in \left\{0,\Delta t,\ldots ,n\Delta t\right\}$$

**Figure 9:**
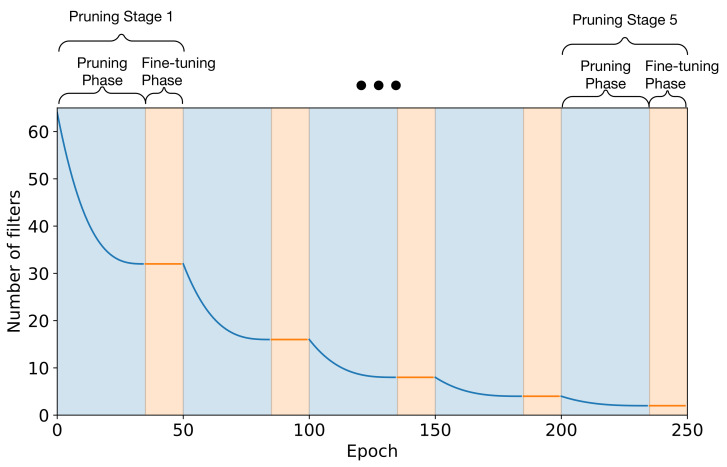
Pruning Schedule Profile.

$s_{f}$ represents sparsity at the end of the pruning phase, i.e., the proportion of filters that need to be pruned, set to 50% for all pruning phases. For the first phase, the filters in each layer are reduced from 64 to 32. The parameter *n* represents the total number of pruning iterations within each pruning phase and is set to 35 epochs for all phases; parameter *t* represents the current pruning iteration in the phase; and the parameter Δt defines the pruning frequency, i.e., how often pruning is allowed. In this case Δt was set to 1 thus allowing pruning at every epoch. It is important to note that unpruned filters between stages and iterations are not re-initialised and maintain their values from the preceding process. Finally, after each pruning stage, the network is fine-tuned for an additional 15 epochs before the execution of the next pruning stage. Using this policy yields the pruning profile shown in Figure 9. Training is executed at a constant learning rate of 1 × 10^−4^ during the pruning phase, as identified by the previous training stage; no learning rate annealing was applied since in every pruning iteration the learning rate is maintained at a constant high value to ensure adequate learning is performed between iterations. However, the fine-tuning phase is executed using the one-cycle training policy described in Section 4.1.1.

Further experimentation to reduce the network memory footprint was performed utilising a mixed precision training policy. Under this policy, the precision of the weights is reduced from single floating point precision (FP32) to half-precision (FP16), the only exception being the trainable parameters for the *BatchNorm* layers which are sustained at FP32 to ensure numerical stability. The pruning procedure described above, is invoked throughout the experiments, starting from the network architecture identified from the hyper-parameter search and trained for a maximum of 50 epochs using the early stopping criterion as described in Section 4.1.1.

**Figure 10:**
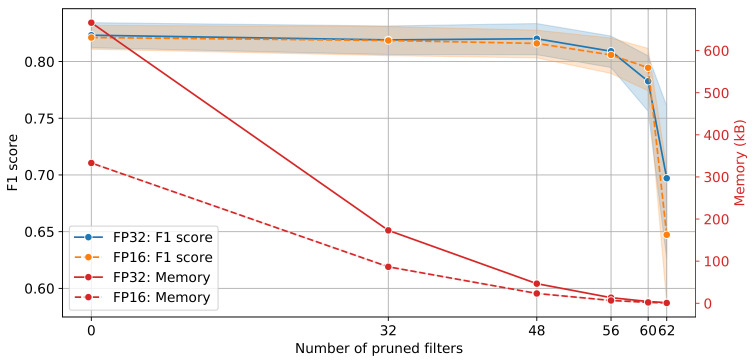
Model performance and memory footprint for different number of filters.

**Figure 11:**
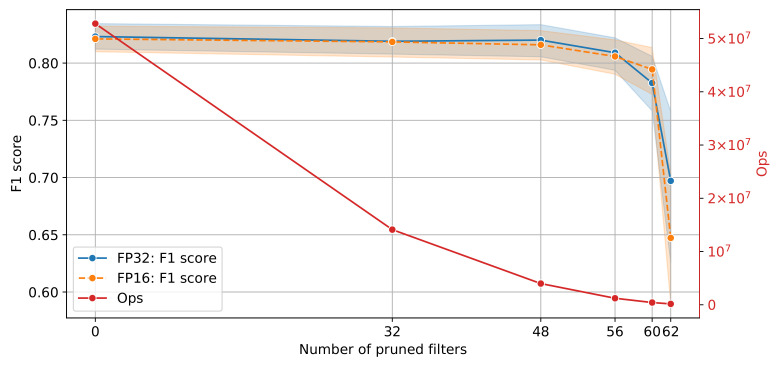
Model performance and network complexity for different number of filters.

All experiments were repeated 5 times. The mean F1 score at the 95% confidence interval on the validation data set, along with the memory reduction for all pruned models for single and half precision are shown in Figure 10. The F1 score performance remains identical with almost half the memory footprint for zero filters pruned. As the number of pruned filters increases to 32, the memory footprint decreases from approximately 666 kB to 173 kB for FP32 without loss in performance. The trend continues until the number of pruned filters reaches 56, after which the performance starts to degrade. Interestingly, the F1 score for 60 pruned filters is slightly higher for FP16 compared to FP32; 0.79 ± 0.02 and 0.78 ± 0.02, respectively. The number of operations (ops) as a function of the performance of the pruned models is shown in Figure 11. The original model requires approximately 52.7 M operations whilst the model with 60 number of pruned filters requires 419.8 k operations.

Inspection of Figure 10 and Figure 11 indicates that the model that balances the trade-off between complexity and classification performance is the 48 pruned filters for both single and half precision. Finally, the model was evaluated on the test set and the F1 score performance achieved was 0.82 for both FP32 and FP16, respectively. Test results are summarised in Table 2 for the original (unpruned) and pruned models for FP32 and FP16 precision. Additionally, the estimated weighted performance in terms of the F1 score for the full test data set, without stratification or balancing, for both pruned models with FP32 and FP16 precision was 0.83.

### 4.3. Practical Implementation on Low-Power Micro-Controllers

One of the key bottlenecks in deploying Deep Learning Neural Networks on wearable animal sensors is the ability to fit the algorithms in terms of memory footprint and computational complexity on constrained micro-controllers. The gulf between the device capabilities and the model complexity has recently narrowed. On one hand, micro-controller memory and computation speed have increased significantly while maintaining low-power operation. For instance, the Cortex-M4 STM32L476RG (ARM, Cambridge, UK) operates at a clock frequency of 80 MHz with 128 kB of SRAM and 1 MB of flash. On the other hand, the pruning analysis presented allows a reduction of the memory footprint and computation complexity of the algorithm without significant loss in performance. These two factors reduces the barrier to providing practical implementations of DL on animal devices.

If the model with 48 pruned filters from Table 2 is considered, its memory requirements are 46.6 kB and 23.3 kB for FP32 and FP16, respectively, which can fit comfortably on the SRAM of the STM32L476RG. Furthermore, according to the manufacturer [52], approximately 9 CPU clock cycles are required to complete an FP32 Multiply-and-accumulate (MACC) operation and given that the number of operations for the 48 pruned filters model is $3.9\times 10^{6}$, a total number of CPU clock cycles is $35.1\times 10^{6}$. Given the CPU clock speed is 80 MHz, 438.75 ms are required to complete a forward pass.

From the perspective of power consumption, the STM32L476RG at 80 MHz clock consumes 10.2 mA while on low-power mode (STOP2) 1.6 A. Given that an inference is performed every 90 s (according to the window length analysis in Section 4.1.3), an active current of 10.2 mA for 438.75 ms and a sleep current draw of 1.6 A for 89.56125 s is required. Hence, the average current consumption is 51.357 A. A typical 3.6 V AA-size battery such as LS14500 [53] 2600 mAh capacity gives an operational lifetime in excess of 5.7 years. Note that this battery life estimate does not consider the current consumption for sampling the accelerometer but typically the average current consumption is in the order of tens of A (for instance, the MPU-6050 [54] has an average current consumption of 70 A sampling at 20 Hz). The use of two battery cells in parallel boosts the available capacity to 4800 mAh without compromising the on-farm implementation.

## 5. Conclusions

CNN implementations have been proven to yield accurate classifications over a range of cattle behavioural states utilising 3-axis accelerometer data from a neck-mounted collar. Hyper-parameter tuning was performed to optimise model architecture, the performance of which was evaluated as a function of time window length. Since the implementation of classification models for on-animal solutions are low-cost with low-power consumption and are thus governed by restrictions in computational resources, full CNN network deployment is challenging since the architectures are relatively complex. Hence, an evaluation of the impact on CNN performance as a function of iterative structured pruning and mixed precision training has been carried out. Results confirm that high performance can still be achieved with significant model reductions that in turn lower the computation complexity and memory footprint requirements significantly. The CNN model with 48 pruned filters is able to classify three cattle behaviours—‘eating’, ‘rumination’ and ‘other’—with an overall F1 score of 0.82 for both FP32 and FP16. This performance is comparable to classic machine learning and deep learning approaches reported in the literature and it is unclear if differences in performance can be attributed to different data sets or model architecture and training methodologies. To aid direct comparisons the data set used in this current study, which is by far the largest reported in the literature, has been made publicly available including raw data and ‘ground truth’ annotations (doi:10.5281/zenodo.4064801). Finally, the proposed model architecture can comfortably fit in the constrained memory of a representative low-power micro-controller such as the ARM Cortex-M4 STM32L476RG, achieving an operational battery-powered lifetime in excess of 5.7 years.

Building on the algorithmic foundation reported within the paper, future research could adopt transfer learning methodologies for the identification of other cattle behaviours of value such as standing, lying and walking. Applicability examination of the proposed algorithmic frameworks to identify walking in free-roaming or grazing animals with the intention of targeting the early identification of the onset of illness (e.g., mastitis, lameness) could be of particular interest for the future study.

## Figures and Tables

**Table 1:** Comparison of published literature on cattle behaviour classification.

	Algorithm	Device	Data Set Size			Ground Truth	Number of Behaviours	Performance (Best Model/Device)			
			Animals	Days	Hours			Acc	Pr	Re	F1
Abell et al. (2017) [18]	DT, RT, RF	C/E/W A	2	3	-	V	4	0.76–0.97	0.02–0.96	0.78–0.94	-
Benaissa et al. (2019) [13]	k-NN, Naive Bayes, SVM	C/P A	16	-	96	HO V	3	0.99	0.96–0.99	0.96–1.00	-
Benaissa et al. (2019) [19]	SVM, DT	C A	10	5	60	HO	3	0.93	0.88–0.98	0.85–0.92	-
Diosdado et al. (2015) [20]	DT, SVM, K-means, HMM	C A	6	6	34	HO	4	-	0.55–0.98	0.77–0.98	-
Dutta et al. (2015) [12]	Ensemble	C A/M GPS	24	10	-	HO	5	0.96	-	0.97	0.89
Gonzalez et al. (2015) [17]	DT	C A/M GPS	58	31	43	HO	5	-	-	0.90	-
Hamilton et al. (2019) [10]	SVM	B A	3	16	181.8	C	2	-	0.83	0.89	0.86
Kasfi et al.(2016) [21]	CNN	C A	22	8	-	HO	2	-	0.82	0.89	0.84
Martiskainen et al. (2009) [22]	SVM	C A	30	30	95.5	V	8	0.84–1.00	0.78	0.00–0.80	-
Peng et al. (2019) [15]	RNN/LSTM	C A/G/M	6	7	420	V	8	0.88	0.88	0.88	0.88
Rahman et al. (2016) [16]	Autoencoder/SVM	C A	22	8	-	HO	9	-	0.40–0.82	0.45–0.95	0.63–0.85
Rahman et al. (2018) [23]	RF	C/H/E A	-	-	-	HO V	3	-	-	-	0.89–0.93
Robert et al. (2009) [24]	DT	P A	15	21	11	V	3	0.98	-	-	-
Smith et al. (2016) [25]	Ensemble	C A	24	8	-	HO	4	-	0.77–0.97	0.69–0.99	0.73–0.98
**Current Study**	**CNN**	**C** **A**	**18**	**62** ^†^	**3460**	**H**	**3**	-	**0.83**	**0.82**	**0.82**

CNN—Convolutional Neural Networks; RNN-LSTM—Recurrent Neural Network with a Long Short-Time Memory; SVM—Support Vector Machines; DT—Decision Tree; RT—random tree; RF—Random Forest; k-NN—k-Nearest Neighbors; C—Collar; H—Halter; E—Ear Tag; P—Pedometer; B—Bolus; W—Wither Tag; A—Accelerometer; G—Gyroscope; M—Magnetometer; V—Video; HO—Human Observation; ^†^ Note: The number of days refers to the unique number of days in the data set. Animals were monitored in blocks and the average observation period per animal was 8.

**Table 2:** A comparison of the performance, memory usage and computation complexity for pruned and original networks for FP16 and FP32.

Pruned Filters	FP Precision	*precision*	*recall*	*F*1 Score	Params	Compression	Operations	Speed-Up	Memory (kB)
0	FP32	0.84	0.82	0.82	170,563	-	$5.2\times 10^{7}$	-	666.2
**48**	**FP32**	**0.83**	**0.82**	**0.82**	**11,923**	**14.30**	$3.9\times 10^{6}$	**13.3**	**46.6**
60	FP32	0.81	0.81	0.81	1063	160.45	$0.4\times 10^{6}$	125.7	4.1
0	FP16	0.83	0.82	0.82	170,563	-	$5.2\times 10^{7}$	-	333.1
**48**	**FP16**	**0.83**	**0.82**	**0.82**	**11,923**	**14.30**	$3.9\times 10^{6}$	**13.3**	**23.3**
60	FP16	0.84	0.83	0.83	1063	160.45	$0.4\times 10^{6}$	125.7	2.0

## Data Availability

The data set is publicly available at https://www.doi.org/10.5281/zenodo.4064801 (accessed on 9 June 2021).

## Appendix A

**Table A1:** Related work: Cattle behaviours.

	Cattle Behaviours
Abell et al. (2017) [18]	Lying, Mounting, Standing & Walking
Benaissa et al. (2019) [13]	Feeding, Lying & Standing
Benaissa et al. (2019) [19]	Feeding, Ruminating & Other
Diosdado et al. (2015) [20]	Feeding, Lying, Standing & Transitions between Standing and Lying
Dutta et al. (2015) [12]	Grazing, Resting, Ruminating, Scratching or urinating & Searching
Gonzalez et al. (2015) [17]	Foraging, Resting, Ruminating, Traveling & Other active behaviors
Hamilton et al. (2019) [10]	Rumination & Non-Rumination
Kasfi et al. (2016) [21]	Grazing & Other
Martiskainen et al. (2009) [22]	Feeding, Lame Walking, Lying, Lying down, Ruminating, Standing, Standing up & Walking normally
Peng et al. (2019) [15]	Feeding, Head butt, Licking salt, Lying, Moving, Ruminating-Lying, Ruminating-Standing & Social licking
Rahman et al. (2016) [16]	Chewing, Grazing, Resting-Lying, Resting-Standing, Ruminating-Lying or Sitting, Ruminating-Standing, Searching, Walking & Other
Rahman et al. (2018) [23]	Grazing, Ruminating & Standing
Robert et al. (2009) [24]	Lying, Standing & Walking
Smith et al. (2016) [25]	Grazing, Resting, Ruminating & Walking
**Current Study**	**Eating, Rumination & Other**
